# Role of Provocable Brugada ECG Pattern in The Correct Risk Stratification for Major Arrhythmic Events

**DOI:** 10.3390/jcm10051025

**Published:** 2021-03-02

**Authors:** Nicolò Martini, Martina Testolina, Gian Luca Toffanin, Rocco Arancio, Luca De Mattia, Sergio Cannas, Giovanni Morani, Bortolo Martini

**Affiliations:** 1 Department of Cardio-Thoraco-Vascular Sciences and Public Health, University of Padua, 35128 Padua, Italy; nicolo.martini2@gmail.com; 2 Cardiac Unit, Alto Vicentino Hospital, 36014 Santorso, Italy; martina.testolina@aulss7.veneto.it (M.T.); gianluca.toffanin@aulss7.veneto.it (G.L.T.); sergio.cannas64@gmail.com (S.C.); giovanni.morani@aulss7.veneto.it (G.M.); 3 Cardiac Unit, Ospedale Umberto Primo, 96100 Siracusa, Italy; arancior@tiscali.it; 4 Cardiac Unit, Treviso Hospital, 32111 Treviso, Italy; dmluca.it@gmail.com

**Keywords:** ajmaline challenge, Brugada syndrome, early repolarization syndrome, arrhythmogenic right ventricular cardiomyopathy, long QT syndrome, hypertrophic cardiomyopathy

## Abstract

The so-called Brugada syndrome (BS), first called precordial early repolarization syndrome (PERS), is characterized by the association of a fascinating electrocardiographic pattern, namely an aspect resembling right bundle branch block with a coved and sometime upsloping ST segment elevation in the precordial leads, and major ventricular arrhythmic events that could rarely lead to sudden death. Its electrogenesis has been related to a conduction delay mostly, but not only, located on the right ventricular outflow tract (RVOT), probably due to a progressive fibrosis of the conduction system. Many tests have been proposed to identify people at risk of sudden death and, among all, ajmaline challenge, thanks to its ability to enhance latent conduction defects, became so popular, even if its role is still controversial as it is neither specific nor sensitive enough to guide further invasive investigations and managements. Interestingly, a type 1 pattern has also been induced in many other cardiac diseases or systemic diseases with a cardiac involvement, such as long QT syndrome (LQTS), arrhythmogenic right ventricular cardiomyopathy (ARVC), hypertrophic cardiomyopathy (HCM) and myotonic dystrophy, without any clear arrhythmic risk profile. Evidence-based studies clearly showed that a positive ajmaline test does not provide any additional information on the risk stratification for major ventricular arrhythmic events on asymptomatic individuals with a non-diagnostic Brugada ECG pattern.

## 1. Introduction

**Figure 1:**
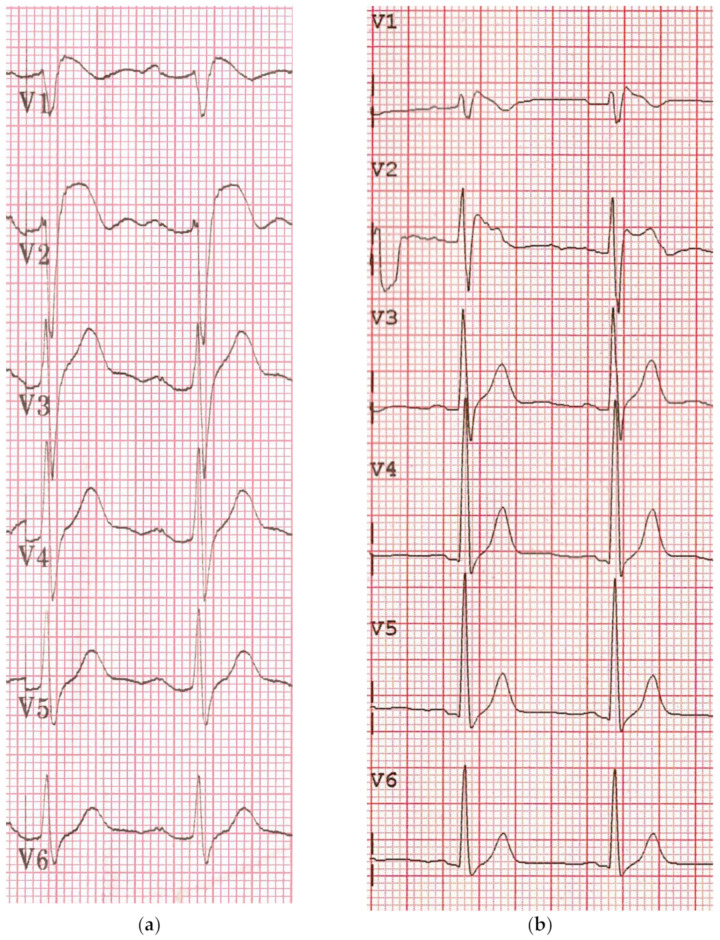
Electrocardiograms (ECGs) showing the precordial leads (V1–V6) of two different patients respectively with a predominant spontaneous type 1 pattern and terminal QRS fragmentations (**a**) and a type 2 pattern (**b**).

The so-called Brugada syndrome (BS), initially called precordial early repolarization syndrome (PERS) [1,2], is mainly characterized by an aspect resembling right bundle branch block, with a coved and sometime upsloping ST segment elevation in the precordial leads (defined as type 1 and 2 patterns) (Figure 1). This electrocardiogram (ECG) has a frequent dynamic behavior, changing from a normal trace to the two types. This ECG pattern that has historically been described as a benign entity [3] has been later rarely associated with arrhythmic cardiac death, and this association has become a popular syndrome nowadays described in more than 5000 papers. The first reports of PERS in a healthy young man [1,2], resuscitated from sudden cardiac death (SCD), was published early in 1988–1989 by Andrea Nava and Bortolo Martini, but became popular a few years later with a different name [4]. Since those old years, many efforts have been made to understand the pathophysiology of this strange ECG (depolarization or repolarization abnormality?), and to identify a correct risk stratification for the affected population. There is robust evidence that the true syndrome, namely the association of a major arrhythmic event and the mentioned ECG pattern, is so rare, but the same cannot be said for the ECG pattern alone. It is noteworthy that many other cardiac or extracardiac conditions, called phenocopies, may share this electrocardiographic pattern [5] but their arrhythmic risk is still unknown, also if they often induce a lot of fear (Brugadaphobia), and an unjustified complex diagnostic and therapeutic management. There has been a rush in medical literature to publish additional patterns to the spontaneous ECG, which could indicate and multiplicate people at risk, but unfortunately at present time, nobody has identified the gold standard, and all this literature is probably nothing more than an anecdotal collection. This paper will discuss, among all the available diagnostic tools, the drug inducible type 1 pattern whose specificity and role in the risk stratification are so controversial, with more doubts than confidences.

## 2. The ECG Patterns

In the worldwide literature, there is an insistent confusion between a syndrome and an ECG pattern alone. A syndrome is the association between symptoms and signs, but the isolated detection of an abnormal ECG does not authorize anybody to make a diagnosis of a lethal condition that is an act that may only induce a severe psychiatric illness in many healthy young people. The true syndrome is so rare, while the isolated type 1 ECG pattern can probably be found in 1 out of thousand people, with the highest prevalence among the Asian population, and type 2 and 3 in up to 3% of healthy people in many reports (Figure 1). A major event can however yearly occur respectively in 0.38 and 0.06% of spontaneous and drug-induced type 1 pattern [6], values like the yearly rate of sudden death in the general population (0.1%).

There is not a typical ECG of the syndrome, that can vary over time, but there is an ECG pattern (defined as type 1), which is more frequent in the true cases of the syndrome. A popular classification in three types, however, does not have a scientific basis as it was only the description of the dynamic behaviour in one single patient [7]. This classification (whence type 3 has now been erased), and the recording of the precordial leads in the upper intercostal spaces has been severely abused. Asymptomatic healthy individuals incidentally found (both spontaneously or drug related) to have one of these three patterns have (and are) been submitted to invasive studies and therapies. Type 1 ECG is indeed a fascinating pattern, that in the true syndromes is more frequently associated to other features, such as a major familial incidence, genetic abnormalities, QRS fractioning, PR interval prolongation, left axis deviation, abnormal electrophysiological investigations, and presence of late potentials, are frequently found in patients with the syndrome [8], but all these findings do not reach enough statistic power to establish any serious evidence-based guideline.

## 3. The Pathophysiological Basis of the Coved Type ECG Pattern

This topic has been the subject of a heavy debate as this pattern, whether inducible or spontaneous, is still poorly understood as well as its prognostic value. Many theories have been proposed, but two major hypotheses have been debated: an organic disease [1,2] vs. a functional abnormality [4]. The first one suggested a conduction delay at the right ventricular outflow tract (RVOT) level as the origin of the ECG pattern and this was retained part of some right ventricular cardiomyopathy as demonstrated by old necropsy study and confirmed by different authors [8,9,10,11]. The disease mainly involves the conduction system (that explains the right bundle branch block (RBBB) pattern, the PR prolongation, the left axis deviation, and the presence of late potentials), and is probably closer to Lev-Lenegre disease (same *SCN5A* genetic abnormality and fibrosis of the conduction system) than to the typical right ventricular cardiomyopathy (ARVC), that has usually different clinical and genetic findings. The ST elevation is a depolarization, rather than a repolarization abnormality, probably reflecting a lesion of the Purkinje network at the RVOT that could also have some embryologic origin [12]. The functional theory instead, proposed that a difference in the action potentials between epicardium and endocardium, (due to ionic channels abnormalities) gives origin to the ECG [13,14]. The organic theory was initially refused [15] and a greater attention was devoted to the functional one that has recently almost been abandoned [16,17]. A re-writing of the controversial history of this syndrome has then been re-proposed [18].

## 4. The Drug Inducible “Brugadophobic” ECG

The syndrome is still retained as one of the leading causes of sudden death and many efforts are made to identify latent asymptomatic carriers at risk. Many tests have been proposed including high precordial recordings, full stomach test, genetic research, signal averaged ECG, vectorcardiography, t-wave alternans, echocardiogram, cardiac magnetic resonance imaging, electrophysiological study, and electroanatomic mapping, but no-one reached enough statistical relevance. Over time, many drug tests have become extremely popular, even if after the occasional identification of a spontaneous type 1 ECG, or maybe a doubtful type 2 or even 3, converted to a type 1 with a pharmacological challenge, a Pandora’s box is sometimes opened. Nowadays, up to 70% of people (mostly asymptomatic) in whom a diagnosis of BS or pattern is made, have been diagnosed by a drug test, performed after a suspicious basal ECG, which only contribute to the creation of the “Brugadophobia”, proposed by Sami Viskin.

Ajmaline is an alkaloid derived from Rauwolfia Serpentina and its effect on cardiac electrogenesis is mediated by the class 1a anti-arrhythmic properties and the ability to induce or enhance a conduction delay [19]. Specifically, an intravenous administration of ajmaline could induce a prolongation of AH and HV intervals, a widening of QRS duration and transient AV blocks [20,21]. Like ajmaline, other antiarrhythmic drugs, particularly class 1c Flecainide, could induce similar abnormalities and are widely used when ajmaline is not available. A similar pattern could also be induced by other cardiological and non-cardiological drugs [22], but this effect is usually due to high or toxic dosages and, at present time, the popular classification of drugs to be avoided is mostly based on their toxic effects and not on a dangerous use of common dosages [23].

**Figure 2:**
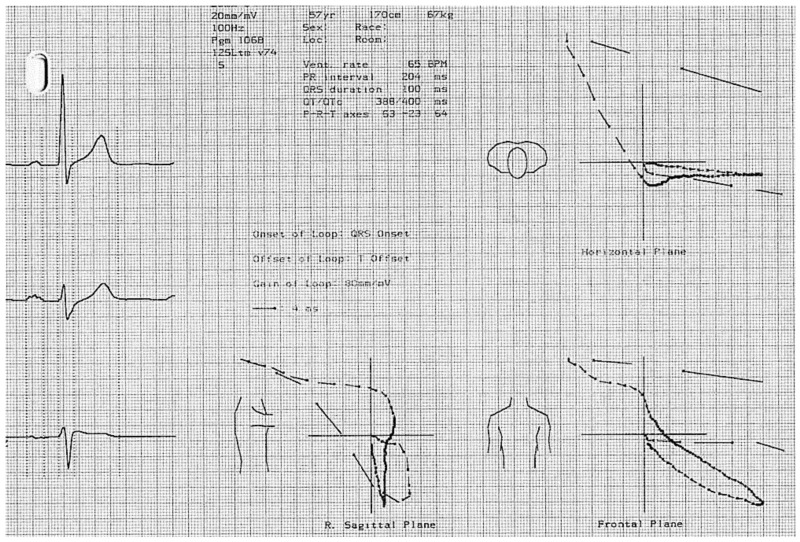
The vectorcardiographic trace of a patient with a spontaneous type 1 pattern. Here, are represented three main loops that indicate the direction of the ventricular activation along three different planes (sagittal, frontal, and horizontal). A right upper-posterior delay is showed, which denotes a late activated portion of the myocardium is the posterior-basal region and the pulmonary conus consistent with a right end conduction disturbance at the right ventricular outflow tract (RVOT).

**Figure 3:**
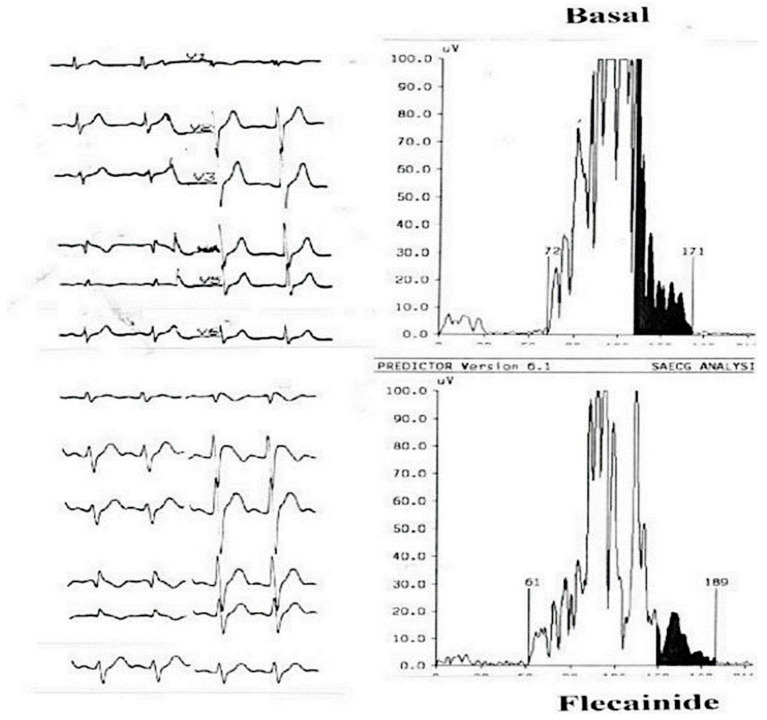
Signal-averaged electrocardiogram (SAECG) of a patient before and after the administration of flecainide. The upper part of the figure shows the baseline ECG and its SAECG trace and no late potentials can be seen. The lower part of the figure belongs to the same patient after the infusion of flecainide: a coved type pattern appears and positive late potentials are induced.

The problem is why ajmaline, flecainide, and the others induce a RVOT delay in many subjects either asymptomatic (up to 5% of normal population treated with flecainide for supraventricular tachycardia, in our experience), or symptomatic with a normal o mildly abnormal ECG. According to Durrer, if we consider the normal activation of the heart, the latest activated portion of the myocardium is the posterior-basal region and the pulmonary conus (RVOT) with different degrees among individuals [24] (Figure 2). Thus, the administration of these drugs could induce or enhance three major conduction delays: a prolongation of HV interval, a right bundle branch block, and a delay at the RVOT-Purkinje system, with the effect of creating an upsloping coved ST segment on the right precordial leads (Type 1) and a vectorcardiographic pattern of upper-posterior terminal QRS delay (Figure 3). The widespread use of these tests is however not totally safe and major ventricular arrhythmias and electromechanical dissociation have been described.

The use of ajmaline introduced by Brugada [25], the flecainide test, and other drugs challenges have different protocols. In Western countries, ajmaline (0.7 mg/kg in 5 min), flecainide (2 mg/kg over 10 min), procainamide (10 mg/kg over 10 min), and propafenone are used. In Japan, pilsicainide, which is a pure Na^+^-channel blocker and a class Ic drug in the Vaughan Williams classification, is usually administrated intravenously at 0.1–1 mg/kg over 10 min. (total 1 mg/kg). No evidence-based differences have been clearly documented between them [26]. Ajmaline was associated to more inducibility of the type 1 pattern compared to procainamide, but no additional prognostic information has been provided, while the pilsicainide challenge showed a major incidence of ventricular arrhythmias, without benefits [27,28]. Despite their diffuse availability and relatively safe profile, major ventricular arrhythmias and electromechanical dissociation have been described as compliances [28,29,30].

Their use to elicit the type 1 ECG pattern is quite sensitive in people with some precordial r1-ST abnormalities but so poorly specific for a clinical syndrome, with a limited predictive value. Unfortunately, the recognition that sodium channel blockers temporarily induce type 1 pattern has let the uncontrolled use of these drugs to unravel the diagnostic ECG; the medical community embraced these new diagnostic tests and at present time, most of the clinical series include asymptomatic people with a drug inducible type 1 ECG, who do not have any syndrome unless proven [31]. As reported by Viskin et al., in Europe, 70% of asymptomatic patients have been diagnosed after a positive ajmaline test, while one of the major reasons for Implantable Cardioverter Defibrillator (ICD) implantation for BS is a positive ajmaline test followed by an inducible ventricular fibrillation (VF) on electrophysiologic studies [31].

The worldwide prevalence of a drug induced type 1 ECG is quite high. It could be elicited in up to 50% of Italian and in 85% of German and French healthy asymptomatic individuals with a type 2 or 3 pattern [32,33,34]. Other series reported a positive response to the Na^+^-channel blocker in 28% of healthy people with a normal baseline ECG and in 2% of people evaluated solely for syncope of unknown origin [35].

The drug induced coved type pattern also showed a good prognosis, as Shimitzu calculated a yearly risk of lethal events of 0.2% [35]. In another analysis performed by Viskin, the risk of a spontaneous VF on asymptomatic patients was only 0.3% per year [36]. A low rate of arrhythmic events was also reported by many other series [37,38], while only a spontaneous coved type pattern seemed to be linked to an increased risk profile [39]. In a more recent metanalysis, Delise calculated a 0.06% annual incidence of lethal events [6].

Other different studies evaluated the role of these drug challenges on the screening of families affected by the syndrome, reporting a low correlation with Sodium Voltage-gated Channel Alpha Subunit 5 (*SCN5A*) gene abnormalities. It is noteworthy that there were more positive tests in asymptomatic subjects than in symptomatic or familial cases, which raises scientific, ethical, and legal questions on these, sometime unsafe, challenges [40,41,42,43]. In these and other published studies, severe limitations were noted because of the low prevalence of the true syndrome, the low event rate, and the short follow up period. The linkage with genetic abnormalities has now been retracted: Brugada initially proposed a 100% correlation between *SCN5A* carriers and the spontaneous or drug inducible ECG pattern, but this assumption was not confirmed, and Priori demonstrated that the test might be negative in as many as 80% of asymptomatic gene carriers [44]. Nobody denied that patients with a drug-induced Brugada-type ECG have a poor prognosis if they also have a history of VF or aborted sudden cardiac death, because the risk of a drug induced Brugada-type ECG is the same as that of a spontaneous Brugada-type ECG [45].

Many concerns raised regarding the invasive electrophysiological study with cardiac stimulation following intra venous injection of the above-mentioned drugs; this can surely increase the number of positive subjects but with a lack in specificity. In recent years, ajmaline has been widely used during epicardial ablation to identify all the areas with a latent conduction disturbance, where to perform extensive ablation. This experimental procedure deserves cautions as there is not consensus in its wide implementation especially in the asymptomatic people submitted to this major invasive procedure.

**Figure 4:**
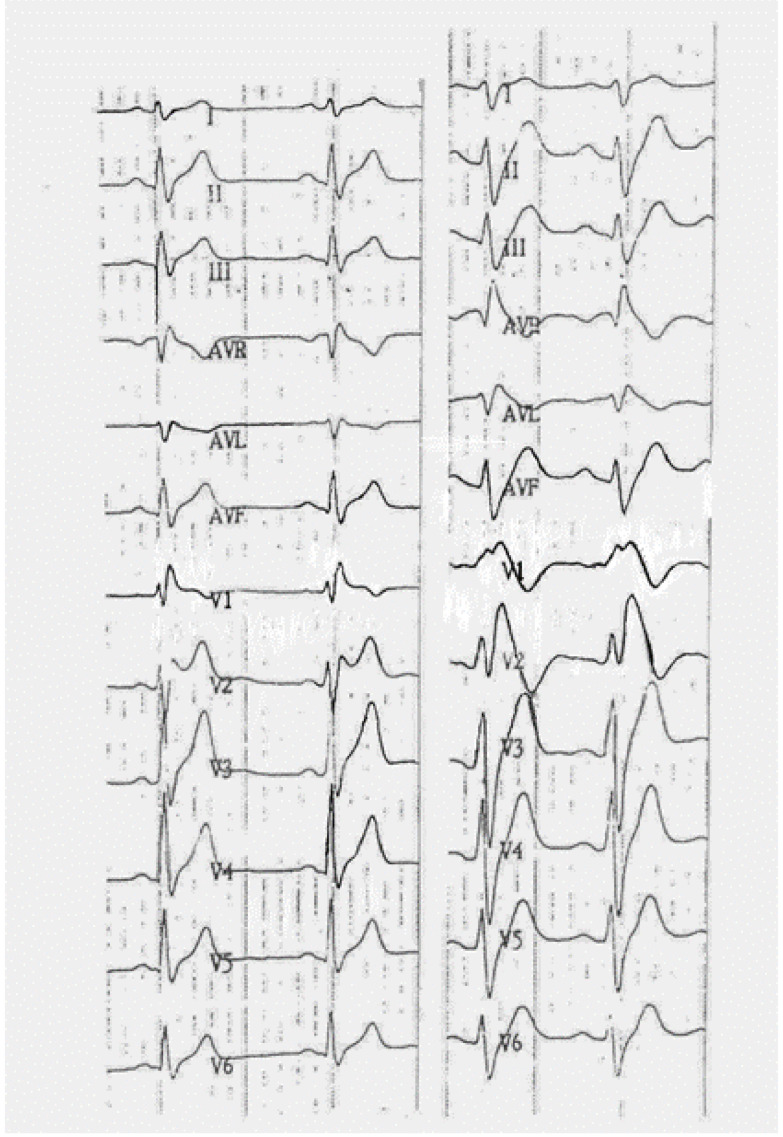
Two 12 leads ECGs of a patient with a familial form of ARVC before (on the left) and after the flecainide challenge (on the right). A massive type 1 pattern appears after the drug administration.

Ajmaline is not specific for BS, and the type 1 pattern was sometimes induced in patients with long QT syndrome (LQT3), but no prognostic values were detected [46]. Patients with ARVC may have similar features (Figure 4). Peters et al. performed the ajmaline challenge on 55 people with a definite diagnosis of ARVC, and 9 of them showed a type 1 pattern [47]. The same author performed the ajmaline test again on 106 patients, 17 of which showed a coved type feature; they were older, mostly of female gender, and symptomatic for syncope [48]. It is of interest that the group of people with a provokable Brugada phenomenon had a low risk of ventricular arrhythmias on follow up, but a higher risk of developing a conduction disease (high degree of atrioventricular block and sinoatrial block) [49].

Ajmaline may disclose the presence of epsilon waves and QRS fragmentation [50,51], like ECG traces in ARVC. This does not mean that BS and ARVC are the same entity but that both can share some RVOT structural abnormalities and consequent similar ECG abnormalities [51].

A positive flecainide testing was also provided in a family (seven members) with an alpha-tropomyosin-induced hypertrophic cardiomyopathy with a very high risk of sudden cardiac death and in 18% of patients affected by myotonic dystrophy and minor ECG anomalies [52,53,54]. Nevertheless, caution should be taken that alpha-tropomyosin gene missense mutation is for certain the cause of a novel overlapping entity with hypertrophic cardiomyopathy and BS. It must be stated that BS may not always be an ion channel dysfunction but may also originate from a myofilament dysfunction that alters Ca^2+^ signaling [53].

## 5. Conclusions

Ajmaline is a drug that has been extensively used throughout the electrophysiological world. Its main role is to enhance various degree of conduction defects [55]. What we can derive from evidence-based study, is that a positive ajmaline test, nowadays, does not provide any clear additional information on the risk stratification for major ventricular arrhythmic events on asymptomatic individuals with a non-diagnostic Brugada ECG pattern, but surely those individuals have a low risk arrhythmic profile. This conclusion reaffirms the wise statements of Sami Viskin: “To conclude, a note of caution: in a recent study simulating screening for Brugada syndrome, as many as 45% of healthy control subjects had minor imperfections in the right precordial leads that could be interpreted as type 2/3 Brugada ECG. We are forced to wonder how often asymptomatic individuals enter a path to rule out BS for the wrong reasons, have a false positive ajmaline test followed by a positive EP study, and faced with the alternative of facing sudden death, end up with an unjustified ICD implantation. Considering the imperfections of the ajmaline test, the study by Tadros has a clear message: A positive ajmaline test does not always mean you have Brugada syndrome” [56].

## Data Availability

No new data were created or analyzed in this study. Data sharing is not applicable to this article.

## References

[1] Nava A., Canciani B., Schiavinato M.L., Martini B., Buja G. (1988). La repolarisation precoce dans le precordiales droites: Trouble de la conduction intraventriculaire droite? Correlations de l’electrocardiographie-vectorcardiographie avec l’electro-physiologie. Mises J. Cardiol..

[2] Martini B., Nava A., Thiene G., Buja G.F., Canciani B., Scognamiglio R., Daliento L., Volta S.D. (1989). Ventricular fibrillation without apparent heart disease: Description of six cases. Am. Hearth J..

[3] Osher H., Wolff L. (1953). Electrocardiographic pattern simulating acute myocardial injury. Am. J. Med. Sci..

[4] Brugada P., Brugada J. (1992). Right bundle branch block, persistent ST segment elevation and sudden cardiac death: A distinct clinical and electrocardiographic syndrome. J. Am. Coll. Cardiol..

[5] Baranchuk A., Nguyen T., Ryu M.H., Femenía F., Zareba W., Wilde A.A.M., Shimizu W., Brugada P., Pérez-Riera A.R. (2012). Brugada Phenocopy: New Terminology and Proposed Classification. Ann. Noninvasive Electrocardiol..

[6] Delise P., Probst V., Allocca G., Sitta N., Sciarra L., Brugada J., Kamakura S., Takagi M., Giustetto C., Calò L. (2017). Clinical outcome of patients with the Brugada type 1 electrocardiogram without prophylactic implantable cardioverter defibrillator in primary prevention: A cumulative analysis of seven large prospective studies. Europace.

[7] Wilde A.A.M., Antzelevitch C., Borggrefe M., Borggrefe M., Brugada J., Brugada R., Brugada P., Corrado D., Hauer R.N.W., Kass R.S. (2002). Proposed diagnostic criteria for the Brugada syndrome. Eur. Heart J..

[8] Martini B., Martini N., Dorantes Sánchez M., Márquez M.F., Zhang L., Fontaine G., Nava A. (2017). Pistas de una enfermedad orgánica subyacente en el síndrome de Brugada. Archivos De Cardiología De México.

[9] Martini B., Corrado D., Nava A., Thiene G., Nava A., Rossi L., Thiene G. (1997). Syndrome of Right bundle branch block, ST segment elevation and sudden death. Evidence of an organic substrate. Arrhythmogenic Right Ventricular Cardiomyopathy/Dysplasia.

[10] Corrado D., Nava A., Buja G., Martini B., Fasoli G., Oselladore L., Turrini P., Thiene G. (1996). Familial cardiomyopathy underlies syndrome of right bundle branch block, ST segment elevation and sudden death. J. Am. Coll. Cardiol..

[11] Corrado D., Basso C., Buja G., Nava A., Rossi L., Thiene G. (2001). Right bundle branch block, right precordial st-segment elevation, and sudden death in young people. Circulation.

[12] Elizari M.V., Levi R., Acunzo R.S., Chiale P.A., Civetta M.M., Ferreiro M., Sicouri S. (2007). Abnormal expression of cardiac neural crest cells in heart development: A different hypothesis for the etiopathogenesis of Brugada syndrome. Hearth Rhythm..

[13] Szél T., Antzelevitch C. (2014). Abnormal Repolarization as the Basis for Late Potentials and Fractionated Electrograms Recorded from Epicardium in Experimental Models of Brugada Syndrome. J. Am. Coll. Cardiol..

[14] Patocskai B., Yoon N., Antzelevitch C. (2017). Mechanisms Underlying Epicardial Radiofrequency Ablation to Suppress Arrhythmogenesis in Experimental Models of Brugada Syndrome. JACC Clin. Electrophysiol..

[15] Brugada P., Brugada J. (1994). Let us do not get confused, please!. G. Ital. Cardiol..

[16] Nademanee K., Veerakul G., Chandanamattha P., Chaothawee L., Ariyachaipanich A., Jirasirirojanakorn K., Likittanasombat K., Bhuripanyo K., Ngarmukos T. (2011). Prevention of Ventricular Fibrillation Episodes in Brugada Syndrome by Catheter Ablation Over the Anterior Right Ventricular Outflow Tract Epicardium. Circulation.

[17] Scheirlynck E., Chivulescu M., Lie Ø.H., Scheirlynck E., Chivulescu M., Øyvind L., Motoc A., Koulalis J., de Asmundis C., Sieira J. (2020). Worse Prognosis in Brugada Syndrome Patients with Arrhythmogenic Cardiomyopathy Features. J. Am. Coll. Cardiol. Electophysiol..

[18] Havakuk O., Viskin S. (2016). A Tale of 2 Diseases: The History of Long-QT Syndrome and Brugada Syndrome. J. Am. Coll. Cardiol..

[19] Obayashi K., Nagasawa K., Mandel W.J., Vyden J.K., Parmley W.W. (1976). Cardiovascular effects of ajmaline. Am. Hearth J..

[20] Padrini R., Piovan D., Javarnaro A., Cucchini F., Ferrari M. (1993). Pharmacokinetics and Electrophysiological Effects of Intravenous Ajmaline. Clin. Pharmacokinet..

[21] Conte G., Levinstein M., Sarkozy A., Sieira J., De Asmundis C., Chierchia G.-B., Di Giovanni G., Baltogiannis G., Ciconte G., Wauters K. (2014). The clinical impact of ajmaline challenge in elderly patients with suspected atrioventricular conduction disease. Int. J. Cardiol..

[22] Letsas K.P., Kavvouras C., Kollias G., Tsikrikas S., Korantzopoulos P., Efremidis M., Sideris A. (2013). Drug-Induced Brugada Syndrome by Noncardiac Agents. Pacing Clin. Electrophysiol..

[23] Postema P.G., Wolpert C., Amin A.S., Probst V., Borggrefe M., Roden D.M., Priori S.G., Tan H.L., Hiraoka M., Brugada J. (2009). Drugs and Brugada syndrome patients: Review of the literature, recommendations, and an up-to-date website (www.brugadadrugs.org). Hearth Rhythm.

[24] Durrer D., Van Dam R.T., Freud G.E., Janse M.J., Meijler F.L., Arzbaecher R.C. (1970). Total Excitation of the Isolated Human Heart. Circulation.

[25] Brugada J., Brugada P., Brugada R. (1996). Ajmaline unmasks right bundle branch block-like and ST segment elevation in V1-V3 in patients with idiopathic ventricular fibrillation. PACE.

[26] Tadros R., Wilde A.A. (2017). Revisiting the sensitivity of sodium channel blocker testing in Brugada syndrome using obligate transmittance. Int. J. Cardiol..

[27] Cheung C.C., Mellor G., Deyell M.W., Ensam B., Batchvarov V., Papadakis M., Roberts J.D., Leather R., Sanatani S., Healey J.S. (2019). Comparison of Ajmaline and Procainamide Provocation Tests in the Diagnosis of Brugada Syndrome. JACC: Clin. Electrophysiol..

[28] Chinushi M., Komura S., Izumi D., Furushima H., Tanabe Y., Washizuka T., Aizawa Y. (2007). Incidence and Initial Characteristics of Pilsicainide-Induced Ventricular Arrhythmias in Patients with Brugada Syndrome. Pacing Clin. Electrophysiol..

[29] Conte G., Sieira J., Sarkozy A., De Asmundis C., Di Giovanni G., Chierchia G.-B., Ciconte G., Levinstein M., Casado-Arroyo R., Baltogiannis G. (2013). Life-threatening ventricular arrhythmias during ajmaline challenge in patients with Brugada syndrome: Incidence, clinical features, and prognosis. Hearth Rhythm.

[30] Moreno J., Magaldi M., Fontanals J., Gómez L., Berne P., Berruezo A., Brugada J. (2014). Use of therapeutic hypothermia and extracorporeal life support after an unusual response to the ajmaline challenge in a patient with Brugada syndrome. J. Cardiol. Cases.

[31] Viskin S., Rosso R., Friedensohn L., Havakuk O., Wilde A.A.M. (2015). Everybody has Brugada syndrome until proven otherwise?. Hearth Rhythm.

[32] Veltmann C., Wolpert C., Sacher F., Mabo P., Schimpf R., Streitner F., Brade J., Kyndt F., Kuschyk J., Le Marec H. (2009). Response to intravenous ajmaline: A retrospective analysis of 677 ajmaline challenges. Eurospace.

[33] Zorzi A., Migliore F., Marras E., Marinelli A., Baritussio A., Allocca G., Leoni L., Perazollo Marra M., Basso C., Buja G. (2012). Should all individuals with a no diagnostic Brugada-electrocardiogram undergo sodium-channel block test?. Heart Rhythm.

[34] Gasparini M., Priori S.G., Mantica M., Napolitano C., Galimberti P., Ceriotti C., Simonini S. (2003). Flecainide Test in Brugada Syndrome: A Reproducible but Risky Tool. Pacing Clin. Electrophysiol..

[35] Shimizu W. (2010). Is This a Philosophic Issue?—Do Patients with Drug-Induced Brugada Type ECG Have Poor Prognosis?. (Pro) Circ. J..

[36] Viskin S., Rosso M. (2017). Read My Lips A Positive Ajmaline Test Does Not Always Mean You Have Brugada Syndrome. JACC.

[37] Sieira J., Ciconte G., Conte G., De Asmundis C., Chierchia G.-B., Baltogiannis G., Di Giovanni G., Saitoh Y., Casado-Arroyo R., Juliá J. (2017). Long-term prognosis of drug-induced Brugada syndrome. Hearth Rhythm.

[38] Nishizaki M., Sakurada H., Yamawake N., Ueda-Tatsumoto A., Hiraoka M. (2010). Low risk for arrhythmic events in asymptomatic patients with drug-induced type 1 ECG. Do patients with drug-induced Brugada type ECG have poor prognosis?. (Con). Circ. J..

[39] Okamura H., Kamakura T., Morita H., Tokioka K., Nakajima I., Wada M., Ishibashi K., Miyamoto K., Noda T., Aiba T. (2015). Risk Stratification in Patients with Brugada Syndrome Without Previous Cardiac Arrest. Circ. J..

[40] Joshi S., Raiszadeh F., Pierce W., Steinberg J.S. (2007). Antiarrhythmic Induced Electrical Storm in Brugada Syndrome: A Case Report. Ann. Noninvasive Electrocardiol..

[41] Morita H., Morita S.T., Nagase S., Banba K., Nishii N., Tani Y., Watanabe A., Nakamura K., Kusano K.F., Emori T. (2003). Ventricular arrhythmia induced by sodium channel blocker in patients with Brugada syndrome. J. Am. Coll. Cardiol..

[42] Antzelevitch C., Brugada P., Brugada J., Brugada R., Towbin J.A., Nademanee K. (2003). Brugada syndrome: 1992-2002: A historical perspective. J. Am. Coll. Cardiol..

[43] Priori S.G., Napolitano C., Schwartz P.J., Bloise R., Crotti L., Ronchetti E. (2000). The Elusive Link between LQT3 and Brugada Syndrome. Circulation.

[44] Priori S.G., Napolitano C., Gasparini M., Pappone C., Della Bella P., Giordano U., Bloise R., Giustetto C., De Nardis R., Grillo M. (2002). Natural History of Brugada Syndrome: Insights for risk stratification and management. Circulation.

[45] Martini B., Zolla C., Guglielmi F., Toffanin G.L., Cannas S., Martini N., Arancio R. (2017). Who is the guilty among these two silent killers?. Heart Rhythm Case Rep..

[46] Hohmann S., Rudic B., Konrad T., Duncker D., König T., Tülümen E., Rostock T., Borggrefe M., Veltmann C. (2016). Systematic ajmaline challenge in patients with long QT 3 syndrome caused by the most common mutation: A multicentre study. Eurospace.

[47] Peters S., Trümmel M., Denecke S., Koehler B. (2004). Results of ajmaline testing in patients with arrhythmogenic right ventricular dysplasia–cardiomyopathy. Int. J. Cardiol..

[48] Peters S. (2008). Arrhythmogenic right ventricular dysplasia-cardiomyopathy and provocable coved-type ST-segment elevation in right precordial leads: Clues from long-term follow-up. Eurospace.

[49] Aras D., Ozeke O., Çay S., Ozcan F., Acar B., Topaloglu S. (2018). Ajmaline-induced epsilon wave: As a potential interim risk factor between the spontaneous- and drug-induced type 1 Brugada electrogram?. Europace.

[50] Martini B., Nava A. (2010). A long-lasting electrocardiographic story. Heart Rhythm.

[51] Corrado D., Zorzi A., Cerrone M., Rigato I., Mongillo M., Bauce B., Delmar M. (2016). Relationship Between Arrhythmogenic Right Ventricular Cardiomyopathy and Brugada Syndrome. Circ. Arrhythmia Electrophysiol..

[52] Mango R., Luchetti A., Sangiuolo R., Ferradini V., Briglia N., Giardina E., Ferre F., Helmer Citterich M., Romeo F., Novelli G. (2016). Next generation sequencing and linkage analysis for the molecular diagnosis of a novel overlapping syndrome charactrized by hypertrophic Cardiomyopathy and typical electrical instability of Brugada syndrome. Circ. J..

[53] Monasky M.M., Ciconte G., Anastasia L., Pappone C. (2017). Commentary: Next Generation Sequencing and Linkage Analysis for the Molecular Diagnosis of a Novel Overlapping Syndrome Characterized by Hypertrophic Cardiomyopathy and Typical Electrical Instability of Brugada Syndrome. Front. Physiol..

[54] Maury P., Audoubert M., Cintas P., Rollin A., Duparc A., Mondoly P., Chiriac A.-M., Acket B., Zhao X., Pasquié J.L. (2014). Prevalence of type 1 Brugada ECG pattern after administration of Class 1C drugs in patients with type 1 myotonic dystrophy: Myotonic dystrophy as a part of the Brugada syndrome. Hearth Rhythm.

[55] Migliore F., Testolina M., Zorzi A., Bertaglia E., Silvano M., Leoni L., Bellin A., Basso C., Thiene G., Allocca G. (2019). First-degree atrioventricular block on basal electrocardiogram predicts future arrhythmic events in patients with Brugada syndrome: A long-term follow-up study from the Veneto region of North-eastern Italy. Europace.

[56] Viskin S., Rosso R. (2017). Read My Lips. JACC Clin. Electrophysiol..

